# How Accurately Can Urologists Predict Eligible Patients for Immediate Postoperative Intravesical Chemotherapy in Bladder Cancer?

**DOI:** 10.3390/diagnostics15151856

**Published:** 2025-07-23

**Authors:** Hüseyin Alperen Yıldız, Müslim Doğan Değer, Güven Aslan

**Affiliations:** 1 Department of Urology, School of Medicine, Bolu Abant İzzet Baysal University, Bolu 14040, Turkey; 2 Department of Urology, Tekirdağ City Hospital, Tekirdağ 59030, Turkey; 3 Department of Urology, School of Medicine, Dokuz Eylül University, İzmir 35330, Turkey

**Keywords:** bladder cancer, cystoscopy, intravesical chemotherapy, prognosis, urothelial carcinoma, visual prediction

## Abstract

**Background/Objectives:** In non-muscle-invasive bladder cancer (NMIBC), the decision for immediate postoperative single-dose intravesical chemotherapy (SI) is based on clinical and presumed pathological features, as a definitive pathology is unknown at the time of surgery. This study aims to assess how accurately urologists can predict the pathological features of bladder tumors based solely on cystoscopic appearance and evaluate their ability to identify patients eligible for SI. **Methods**: A total of 104 patients with bladder masses were included. Seven senior urologists and four residents participated. Before transurethral resection, both groups predicted tumor stage, grade, and the presence of carcinoma in situ (CIS). Resident predictions were collected for all 104 patients, while senior predictions were collected for 72 patients. Based on these predictions, patient eligibility for SI was determined according to the EAU NMIBC guidelines. After final pathology reports, risk scores were recalculated and compared with the surgeons’ predictions. Cohen’s Kappa (κ) coefficient was used to assess agreement between predictions and pathology. Positive and negative predictive values were also calculated for both groups. **Results**: Strong agreement with final pathology could not be demonstrated for stage, grade, or CIS for either group. Urology residents’ predictions were slightly more accurate than those of senior urologists. Overall, 19.4% (14/72) (based on senior urologists’ predictions) and 18.2% (19/104) (based on resident predictions) of patients were misclassified and either overtreated or undertreated. **Conclusions**: Cystoscopic visual prediction alone is insufficient for determining eligibility for immediate postoperative intravesical chemotherapy, regardless of the urologist’s experience. More objective criteria are needed to improve the selection of appropriate patients for SI.

## 1. Introduction

Bladder cancer is the 7th most frequently diagnosed cancer among men worldwide, with its incidence decreasing to 11th when both sexes are considered [1]. Recent epidemiological data reported approximately 550,000 new cases globally in 2022, with non-muscle-invasive bladder cancer (NMIBC) constituting approximately 75% of these diagnoses [1,2]. Given the significant burden of bladder cancer, accurate diagnosis and effective treatment strategies remain critical areas of clinical and research interest.

The standard diagnostic and initial therapeutic approach for suspected bladder cancer involves cystoscopy followed by transurethral resection of bladder tumors (TURBTs) [2]. Immediate postoperative intravesical chemotherapy (SI) is increasingly recognized as an essential adjunctive treatment recommended for patients with low- to intermediate-risk NMIBC tumors, significantly reducing recurrence rates by approximately 14% within five years [3,4]. This practice is strongly endorsed by the latest guidelines from both the European Association of Urology (EAU) and the American Urological Association (AUA) [2,4].

Risk stratification models, such as the European Organisation for Research and Treatment of Cancer (EORTC) and the Spanish Urological Club for Oncological Treatment (CUETO), are frequently used to predict prognosis and guide treatment decisions [5,6]. These models are based on cystoscopic findings and the pathological features of the tumor, which are essential in determining appropriate treatment. While factors such as tumor size, number of tumors, and prior recurrence rates are relatively objective, others like the T category, tumor grade, and presence of carcinoma in situ (CIS) are more subjective and dependent on the surgeon’s intraoperative assessment. With this decision, the patient will either receive a single dose of chemotherapy that will benefit the recurrence of the disease or will be exposed to an unnecessary drug administration with its associated risks. This decision will be made by the urologist during cystoscopy with visual prediction. Therefore, the concordance of this decision with the final pathology is crucial.

Despite advances in diagnostic imaging such as multiparametric MRI and advanced cystoscopy techniques including narrow-band imaging (NBI) and photodynamic diagnosis (PDD), traditional cystoscopy remains the most accessible diagnostic tool in clinical practice [7,8]. While cystoscopy is generally reliable for distinguishing malignant from benign bladder lesions, recent studies highlight variability in its accuracy for predicting detailed histopathological features, particularly tumor stage and grade. For instance, recent research demonstrated moderate cystoscopic accuracy in predicting muscle invasion, with significant potential for misclassification [9]. Further studies have identified limitations of cystoscopy in accurately detecting and grading all tumors, suggesting the necessity of supplementary diagnostic tools [10].

To address these limitations, artificial intelligence (AI)-assisted cystoscopic analyses have recently been explored, showing promising results in improving diagnostic accuracy and reducing inter-observer variability [11,12,13,14]. These developments highlight the potential for integrating advanced technologies into traditional practices to enhance diagnostic precision.

Considering the critical role immediate postoperative intravesical chemotherapy plays in patient outcomes, the accuracy of intraoperative visual predictions significantly impacts clinical decision-making and patient management. Errors in visual predictions can lead to overtreatment or undertreatment, each carrying significant clinical and economic consequences. Overtreatment exposes patients to unnecessary chemotherapeutic risks and increases healthcare costs, whereas undertreatment may raise the risk of recurrence, progression, and subsequent morbidity [15,16].

Given these critical considerations, systematically evaluating how accurately urologists can predict bladder tumor pathology based solely on cystoscopic appearance remains imperative. Furthermore, assessing whether surgical experience influences predictive accuracy could inform training programs and clinical guidelines, potentially reducing errors in patient management.

Therefore, this study aims to assess the accuracy of visual cystoscopic predictions by senior urologists and urology residents regarding tumor stage, grade, and the presence of carcinoma in situ (CIS). Additionally, it evaluates the impact of these predictions on eligibility determination for immediate postoperative intravesical chemotherapy according to current EAU guidelines. Addressing this research gap could significantly enhance clinical practices, training strategies, and ultimately, patient outcomes in bladder cancer management.

## 2. Materials and Methods

**Figure 1:**
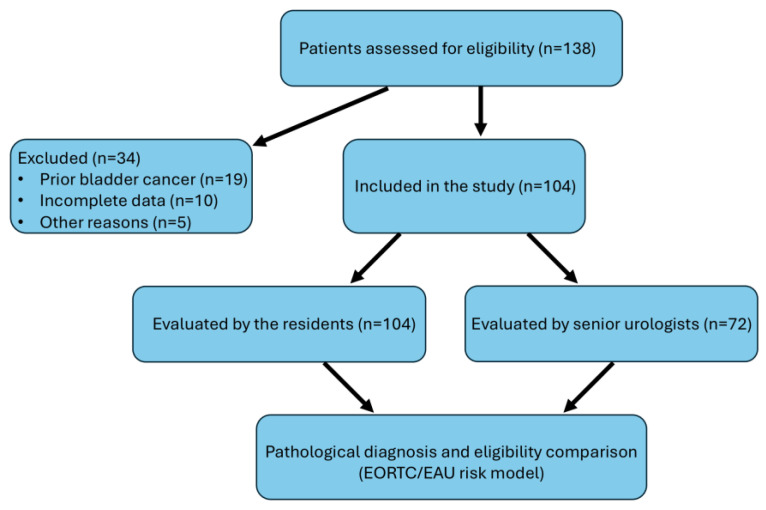
Flowchart of the study population.

This prospective observational study initially evaluated 138 consecutive patients admitted to our clinic with suspected bladder masses. Of these, 34 patients were excluded from the final analysis: 19 due to a prior history of bladder cancer, 10 due to incomplete clinical or pathological data, and 5 due to the presence of acute urinary tract infection or gross hematuria that precluded adequate cystoscopic visualization. After applying these criteria, 104 patients were included in the study for further analysis (Figure 1).

Inclusion criteria:

- Age ≥ 18 years.
- Newly diagnosed bladder mass detected by imaging studies or cystoscopy.
- No prior history of bladder cancer.
- Ability to provide informed consent.

Exclusion criteria:

- Previous history of bladder cancer or bladder cancer treatment.
- Recurrent bladder tumors.
- Patients with incomplete clinical records or missing follow-up information.
- Patients unable or unwilling to provide informed consent.
- Pregnancy.
- Presence of acute urinary tract infection at the time of evaluation.

All eligible patients underwent standard white-light rigid cystoscopy conducted by experienced urologists and supervised residents. Prior to transurethral resection of the bladder tumor (TURBT), urologists were asked to visually assess and record predictions regarding the tumor stage (Ta, T1, T2), grade (low/high), and the presence of carcinoma in situ (CIS). Senior urologists provided predictions for 72 patients, while residents provided predictions for all 104 patients.

Immediately following visual assessment, TURBT was performed according to standardized surgical protocols, ensuring complete resection of all visible lesions using a resectoscope (Karl Storz, Tuttlingen, Germany). Specimens collected were systematically labeled and sent to pathology for analysis. Pathological examination was conducted by board-certified uropathologists blinded to the visual predictions of the surgeons. Tumor staging was classified based on the 2017 TNM classification, and tumor grading followed the 2004/2016 WHO guidelines [17,18].

Agreement between cystoscopic visual predictions and pathological findings was evaluated using Cohen’s Kappa coefficient (κ), with interpretation as follows: ≤0 indicating no agreement, 0.01–0.20 poor, 0.21–0.40 fair, 0.41–0.60 moderate, 0.61–0.80 substantial, and 0.81–1.00 almost perfect agreement [19]. Positive predictive value (PPV), negative predictive value (NPV), sensitivity, and specificity were calculated for each observer group. Residents and senior urologists were analyzed as collective groups, with each group treated as a single composite rater. A post hoc power analysis was performed to evaluate whether the study sample was sufficient to detect a clinically meaningful difference in agreement. A minimum of 87 patients is required to distinguish between fair (κ = 0.4) and moderate (κ = 0.6) levels of agreement with 80% power and a 0.05 significance level, assuming two raters per subject [20]. Given that our sample included 72 patients in the senior group and 104 in the resident group, both cohorts fall within an acceptable margin to detect such differences when inter-rater observations and simplified prediction assessments are accounted for.

EAU risk scores and EORTC recurrence scores were calculated for all patients predicted to have NMIBC (Tables S1 and S2). Patients predicted to be in the low- to intermediate-risk group and with an EORTC recurrence score of less than 5 were considered eligible for SI. After pathological examination, risk and EORTC recurrence scores were recalculated based on the final pathology report and compared with the surgeons’ predictions to identify misclassifications, overtreatment, or undertreatment.

## 3. Results

The mean age of the patients was 67.6 ± 10.7; 84.6% were male, and 15.5% were female. A total of 16 (15.3%) patients were diagnosed with muscle-invasive bladder cancer (MIBC) and 88 (84.6%) with NMIBC on pathological analysis. A total of 59 (56.7%) patients were evaluated as high-grade and 45 (43.2%) patients as low-grade on pathological analysis. Detailed demographic and clinical characteristics are presented in Table 1. To evaluate potential bias arising from the absence of senior urologist predictions in 32 patients, we compared demographic and tumor characteristics between patients with and without senior assessments. Variables compared included age, gender, pathological stage, grade, and presence of CIS. No statistically significant differences were observed between the groups (*p* > 0.05 for all variables).

When comparing senior urologists’ predictions with pathological assessment, there was a fair correlation for stage, a moderate correlation for grade, and a poor correlation for the presence of CIS (kappa: 0.333, 0.528, 0.180, respectively) (Table 2). As we look at the agreement between residents’ predictions and pathological assessment, there was a fair correlation for stage, moderate correlation for grade, and fair correlation for presence of CIS (kappa: 0.393, 0.574, 0.228, respectively) (Table 3). Residents’ predictions were slightly more accurate than senior urologists’ for stage, grade, and the presence of CIS (Table 4).

All surgeons were more likely to overstage and overgrade bladder cancer. The likelihood of predicting NMIBC as MIBC was 68.4% for seniors and 64.2% for residents. Conversely, the likelihood of predicting MIBC as NMIBC was 9.4% (5/53) and 7.8% (6/76). The likelihood of predicting low-grade tumors as high-grade was 25.4% (13/51) and 27.4% (17/62). Whereas likelihood of predicting high-grade tumors as low-grade was 14.2% (3/21) and 12.5% (4/32) for seniors and residents, respectively.

When tumor grade, stage, and presence of CIS were predicted together, 30/72 (41.6%) cases were in full agreement with the pathological evaluation for senior urologists and 46/104 (44.2%) for resident urologists.

Both seniors and residents could predict low-stage and -grade tumors more accurately than the tumors with higher stage and grade (Table 4).

Senior urologists predicted 20 of 72 patients as eligible for SI, with 15% (3/20) of these patients deemed ineligible after pathological evaluation. Among the 52 patients predicted to be ineligible, 21.1% (11/52) were determined to be eligible after pathology. Overall, 19.4% (14/72) of patients were overtreated or undertreated based on surgeons’ predictions.

Among residents, 31 of 104 patients were predicted as eligible for SI, with 12.9% (4/31) found to be ineligible after pathology. Among the 73 predicted ineligible, 20.5% (15/73) were deemed eligible after pathology. In total, 18.2% (19/104) of patients were either overtreated or undertreated.

## 4. Discussion

Accurately predicting the stage and grade of bladder cancer during cystoscopy is essential for guiding clinical decisions, including the administration of postoperative intravesical chemotherapy (SI). Several meta-analyses have demonstrated the benefits of SI in reducing recurrence rates in non-muscle-invasive bladder cancer (NMIBC), highlighting the importance of early and accurate risk stratification [3,15,21]. However, as the definitive stage and grade of the tumor are not available at the time of surgery, urologists must rely on visual assessments during cystoscopy to determine patient eligibility for SI.

In examining our results according to pathological parameters separately, urologists were able to predict low-stage and low-grade tumors better than pT1, pT2, and high-grade cancers. However, in the correlation analysis, strong agreement with the pathological report could not be demonstrated with any of the stages, grades, and presence of CIS.

We initially hypothesized that more-experienced urologists would be better at predicting these pathological features. Contrary to our expectations, we found that residents were slightly more accurate than senior urologists in their predictions. One possible explanation for this finding is that residents, being involved in every operation during their training, may have more recent and hands-on experience compared to senior urologists, who primarily supervise procedures and may not be involved in as many day-to-day surgeries. This suggests that recent and frequent experience in the operating room may play a larger role than accumulated years of practice in making accurate intraoperative assessments.

The accuracy of cystoscopy in predicting histopathological features of bladder tumors has been a subject of debate in the literature. It has been previously shown that urologists can distinguish malignant lesions from benign lesions quite accurately [22]. Also, there are several studies with conflicting results investigating accuracy of cystoscopy regarding prediction of the histological features of the tumor [22,23,24,25]. Cina et al. reported that while urologists achieved 100% positive and negative predictive values in differentiating between benign and malignant lesions, they could not accurately predict deeper tissue involvement or the tumor stage [22]. Similarly, Steffens et al., in a multicenter study, found that cystoscopy had a positive predictive value (PPV) of only 52% for muscle-invasive bladder cancer (MIBC) and an impressive negative predictive value (NPV) of 95%, irrespective of the surgeon’s experience. These findings resonate with our results, where surgeons frequently overestimated tumor stage, leading to overtreatment in a significant number of patients [24].

Contrary to these findings, During et al. reported higher accuracy in predicting MIBC, with a PPV of 78.4% and an NPV of 95.7% [26]. Likewise in a recent study, it is reported that positive predictive values for low- and high-grade cancers were 85.8% and 71.3%, respectively; and non–muscle-invasive and muscle-invasive cancers were predicted accurately in 93.4% and 85.2% patients, respectively [27].

These studies suggest that while there is some variability in the reported accuracy of cystoscopic visual assessment, it remains a limited tool for reliably predicting tumor stage and grade, especially for more advanced or invasive cancers. None of these studies, however, focused on patient selection for immediate postoperative chemotherapy, which is a unique aspect of our investigation.

Our study highlights a critical issue: a significant proportion of patients who may not benefit from SI are being overtreated, which can lead to unnecessary morbidities and increased healthcare costs. Conversely, some patients who might benefit from early chemotherapy are being overlooked, potentially increasing their risk of disease recurrence. The variability in urologists’ predictions, especially regarding higher-grade and higher-stage tumors, underscores the need for more objective and reliable intraoperative assessment tools.

While tumor size and the number of tumors have been identified as predictors of recurrence in EORTC trials, the decision to administer SI often relies heavily on the urologist’s estimation of tumor stage, grade, and CIS presence—factors that, as our findings suggest, are prone to inaccuracy [5].

To improve diagnostic accuracy, researchers have increasingly investigated the use of artificial intelligence (AI) in bladder cancer diagnosis. Deep learning models have demonstrated high precision in classifying bladder tumor images obtained during cystoscopy, outperforming conventional visual assessments in detecting both NMIBC and MIBC [11]. These models can identify subtle features not easily perceived by the human eye, leading to better diagnostic outcomes. In a recent study, ensemble deep learning techniques were able to classify tumor grade and invasiveness with high sensitivity and specificity based on cystoscopic images alone [13].

Beyond cystoscopy, AI has also been applied in conjunction with advanced imaging modalities such as multiparametric MRI. The VI-RADS scoring system, supported by machine learning models, has proven effective in differentiating NMIBC from MIBC, improving the staging process preoperatively [28,29]. Studies suggest that radiomics combined with AI can provide reproducible and objective assessments, further reducing inter-observer variability [28,29].

Additionally, clinical decision support systems (CDSSs) have emerged as valuable tools to aid surgeons during real-time decision-making. By integrating visual assessments, patient history, and guideline-based algorithms, CDSS platforms can enhance treatment accuracy. Studies have shown that CDSSs improve adherence to evidence-based practices and reduces variability among providers [30,31,32].

Despite these advancements, the real-world use of immediate intravesical chemotherapy remains limited. In a recent US-based analysis, only 5.6% of patients undergoing TURBT received a postoperative instillation, far below the guideline-recommended rate [33]. Our study further underscores this challenge by revealing a 19% misclassification rate in SI eligibility when relying solely on intraoperative visual assessment. To reduce such errors and improve adherence to guidelines, the adoption of enhanced intraoperative tools is warranted. Technologies such as Narrow Band Imaging (NBI), intraoperative frozen section analysis, and AI-assisted decision support systems may improve the accuracy of tumor characterization in real-time, thereby facilitating appropriate SI use. Future studies should explore the feasibility, accuracy, and cost-effectiveness of integrating such tools into routine clinical workflows.

Reasons for this discrepancy include surgeon uncertainty about pathological risk, concerns about side effects, and logistical challenges. Enhanced intraoperative assessment tools—including AI-powered decision support—could reduce such uncertainty and improve compliance with treatment guidelines.

There are several limitations to our study that should be acknowledged. First, we were unable to obtain predictions from senior urologists for 32 patients due to their absence in the operating room for these specific cases. This was due to the high surgical volume in our clinic, which may have impacted the overall comparison between residents and senior urologists. Moreover, the identity of the individual rater was not recorded in either group. As a result, multi-rater agreement measures such as Fleiss’ kappa could not be applied, and individual-level κ values could not be calculated. Both residents and senior urologists were analyzed as collective groups, each treated as a single composite rater. This approach does not account for potential variability within each group and limits the ability to assess inter-individual predictive performance.

Additionally, while T1 tumors are known to have a high risk of understaging (reported to be between 8 and 49% in the literature), we based our analysis on the initial pathological reports and did not evaluate repeat transurethral resection of the bladder (TURB) reports [34,35]. As repeat TURB is often recommended to accurately stage T1 tumors, some of our cases may have been pathologically understaged. However, this does not significantly affect our conclusions regarding SI, as patients with T1 tumors were not the primary target for this treatment strategy in our study.

## 5. Conclusions

Although cystoscopy is a valuable diagnostic tool for bladder urothelial carcinoma, it is not reliable for accurately predicting the stage and grade of tumors, regardless of the surgeon’s experience. Cystoscopic findings cannot substitute for histopathological analysis in managing the disease or predicting patient prognosis. Visual assessments made during cystoscopy are insufficient for determining the need for immediate postoperative intravesical chemotherapy. Incorporating more objective criteria—including artificial intelligence, imaging biomarkers, and decision support algorithms—may help urologists more accurately assess tumor risk and reduce treatment errors. Future research should focus on integrating these tools into routine clinical workflows, potentially transforming the standard of care for NMIBC patients.

## Figures and Tables

**Table 1:** Demographic and clinical characteristics of the study population.

Characteristics	Values
Total patients	104
Mean age (years ± SD)	67.6 ± 10.7
Male, n (%)	88 (84.6%)
Female, n (%)	16 (15.4%)
Pathological stage, n (%) Ta T1 T2 (MIBC)	44 (42.3%) 44 (42.3%) 16 (15.4%)
Pathological grade, n (%) Low grade High grade	45 (43.2%) 59 (56.7%)
Carcinoma in situ (CIS), n (%) Present Absent	20 (19.2%) 84 (80.8%)

**Table 2:** Association between pathological and predicted tumor features by senior urologists.

		Pathological Stage					
		Ta (29)		T1 (32)		T2 (11)	
Predicted Stage	Ta (22)	19		3		0	
	T1 (31)	10		16		5	
	T2 (19)	0		13		6	
Kappa 0.333 (0.115–0.551 CI 95%) (fair correlation)							
		Pathological Grade					
		Low-Grade (31)			High-Grade (41)		
Predicted Stage	Low-Grade (21)	18			3		
	High-Grade (51)	13			38		
Kappa 0.528 (0.332–0.724 CI 95%) (moderate correlation)							
		Pathological CIS					
		Positive (14)			Negative (58)		
Predicted CIS	Positive (11)	4			7		
	Negative (61)	10			51		
Kappa 0.180 (−0.047–0.407 CI 95%) (poor correlation)							

**Table 3:** Association between pathological and predicted tumor features by resident urologists.

		Pathological Stage					
		Ta (44)		T1 (44)		T2 (16)	
Predicted Stage	Ta (33)	29		4		0	
	T1 (43)	13		24		6	
	T2 (28)	2		16		10	
Kappa 0.393 (0.216–0.570 CI 95%) (fair correlation)							
		Pathological Grade					
		Low-Grade (45)			High-Grade (59)		
Predicted Stage	Low-Grade (32)	28			4		
	High-Grade (72)	17			55		
Kappa 0.574 (0.417–0.731 CI 95%) (moderate correlation)							
		Pathological CIS					
		Positive (20)			Negative (84)		
Predicted CIS	Positive (18)	7			11		
	Negative (86)	13			73		
Kappa 0.228 (0.041–0.415 CI 95%) (fair correlation)							

**Table 4:** Positive predictive values for urology specialists and residents.

	Specialists		Residents	
	PPV (95% CI)	NPV (95% CI)	PPV (95% CI)	NPV (95% CI)
Ta	86.36% (67.33–95.11%)	80% (70.64–86.93%)	87.88% (73.32–95.03%)	78.87% (71.11–84.99%)
T1	51.61% (38.59–64.42%)	60.98% (50.62–70.43%)	55.81% (44.38–66.66%)	67.21% (58.69–74.73%)
T2 (MIBC)	31.58% (18.29–48.76%)	90.57% (83.22–94.89%)	35.71% (24.09–49.31%)	92.11% (86–95.68%)
Ta/T1 (NMIBC)	90.57% (83.22–94.89%)	31.58% (18.29–48.76%)	92.11% (86.00–95.68%)	35.71% (24.09–49.31%)
High Grade	74.51% (65.69–81.69%)	85.71% (65.97–94.89%)	76.39% (68.85–82.57%)	87.5% (72.57–94.88%)
Low Grade	85.71% (65.97–94.89%)	74.51% (65.69–81.69%)	87.50% (72.57–94.88%)	76.39% (68.85–82.57%)
CIS	36.36% (16.24–62.75%)	83.61% (78.32–87.8%)	38.89% (22.02–58.92%)	84.88% (80.11–88.67%)

## Data Availability

The datasets generated and analyzed during the current study are not publicly available due to ethical and privacy concerns but are available from the corresponding author on reasonable request.

## Supplementary Materials

The following supporting information can be downloaded at: https://www.mdpi.com/article/10.3390/diagnostics15151856/s1, Table S1. EORTC risk scores for recurrence and progression [5]; Table S2. EAU NMIBC Risk Group Classification [2].

## Abbreviations

The following abbreviations are used in this manuscript:

NMIBC	Non-Muscle-Invasive Bladder Cancer
MIBC	Muscle-Invasive Bladder Cancer
SI	Single, Immediate postoperative intravesical chemotherapy
CIS	Carcinoma In Situ
TUR	Transurethral Resection
EAU	European Association of Urology
EORTC	European Organisation for Research and Treatment of Cancer
CUETO	Spanish Urological Club for Oncological Treatment
WHO	World Health Organization
VI-RADS	Vesical Imaging-Reporting and Data System
CT	Computed Tomography
MRI	Magnetic Resonance Imaging
AI	Artificial Intelligence
CDSS	Clinical Decision Support System
