# Supplementary material for: How Accurately Can Urologists Predict Eligible Patients for Immediate Postoperative Intravesical Chemotherapy in Bladder Cancer?
Source: Diagnostics (Basel). 2025 Jul 23;15(15):1856. doi: 10.3390/diagnostics15151856 (PMC12346383; doi:10.3390/diagnostics15151856)
Supplement: Supplementary file 1 [file diagnostics-15-01856-s001.zip › diagnostics-3709505-supplementary.pdf]

Table S1. *EORTC risk scores for recurrence and progression [5].*

Factor	Category	Recurrence Score	Progression Score
Number of tumors	Single	0	0
	2 to 7	3	3
	≥8	6	3
Tumor size	<3 cm	0	0
	≥3 cm	3	3
Prior recurrence rate	Primary	0	0
	≤1 recurrence/year	2	2
	>1 recurrence/year	4	2
T category	Ta	0	0
	T1	1	4
CIS	No	0	0
	Yes	1	6
Grade	G1	0	0
	G2	1	0
	G3	2	5
Total Score Range		0–17	0–23

Table S2. *EAU NMIBC Risk Group Classification [2].*

Risk Group	Definition
Low Risk	• A primary, single, TaT1 LG/G1 tumour < 3 cm in diameter without CIS in a patient ≤ 70 years<br>• A primary Ta LG/G1 tumour without CIS with at most ONE of the additional clinical risk factors
Intermediate Risk	• Patients without CIS who are not included in either the low-, high-, or very high-risk groups
High Risk	• All T1 HG/G3 without CIS, EXCEPT those included in the very high-risk group<br>• All CIS patients, EXCEPT those included in the very high-risk group<br><br>Stage, grade with additional clinical risk factors:<br>• Ta LG/G2 or T1G1, no CIS with all 3 risk factors<br>• Ta HG/G3 or T1 LG, no CIS with at least 2 risk factors<br>• T1G2 no CIS with at least 1 risk factor
Very High Risk	Stage, grade with additional clinical risk factors:<br>• Ta HG/G3 and CIS with all 3 risk factors<br>• T1G2 and CIS with at least 2 risk factors<br>• T1 HG/G3 and CIS with at least 1 risk factor<br>• T1 HG/G3 no CIS with all 3 risk factors
